# Feasibility study on effect and stability of adaptive radiotherapy on kilovoltage cone beam CT

**DOI:** 10.2478/v10019-011-0024-5

**Published:** 2011-07-20

**Authors:** Poonam Yadav, Velayudham Ramasubramanian, Bhudatt R. Paliwal

**Affiliations:** 1 Department of Human Oncology, University of Wisconsin, Madison, WI, USA; 2 Department of Medical Physics, University of Wisconsin, Madison, WI, USA; 3 Vellore Institute of Technology University, Vellore, Tamil Nadu, India

**Keywords:** cone-beam computerized tomography, kV CBCT, CT to density table, electron density phantom, adaptive planning

## Abstract

**Background:**

We have analyzed the stability of CT to density curve of kilovoltage cone-beam computerized tomography (kV CBCT) imaging modality over the period of six months. We also, investigated the viability of using image value to density table (IVDT) generated at different time, for adaptive radiotherapy treatment planning. The consequences of target volume change and the efficacy of kV CBCT for adaptive planning issues is investigated.

**Materials and methods.:**

Standard electron density phantom was used to establish CT to electron density calibrations curve. The CT to density curve for the CBCT images were observed for the period of six months. The kV CBCT scans used for adaptive planning was acquired with an on-board imager system mounted on a “Trilogy” linear accelerator. kV CBCT images were acquired for daily setup registration. The effect of variations in CT to density curve was studied on two clinical cases: prostate and lung.

**Results:**

The soft tissue contouring is superior in kV CBCT scans in comparison to mega voltage CT (MVCT) scans. The CT to density curve for the CBCT images was found steady over six months. Due to difficulty in attaining the reproducibility in daily setup for the prostate treatment, there is a day-to-day difference in dose to the rectum and bladder.

**Conclusions:**

There is no need for generating a new CT to density curve for the adaptive planning on the kV CBCT images. Also, it is viable to perform the adaptive planning to check the dose to target and organ at risk (OAR) without performing a new kV CT scan, which will reduce the dose to the patient.

## Introduction

The ultimate goal of radiotherapy is to deliver a sufficient dose to the target (tumor) region while sparing the healthy tissues around target so that successful target cell killing can be achieved with minimum toxicity to the organ at risk (OAR). Limited patient motion also helps to assure that the dose delivered during the treatment is close to the dose computed on initial kVCT images used for the treatment planning. Modern irradiation techniques such as stereotactic radiation and intensity-modulated radiotherapy (IMRT) are capable of generating the complex dose distribution with high dose areas firmly conformed to the target volume.^1^–^3^ The sparing of surrounding normal tissue is efficiently achieved if the patient is accurately positioned on the treatment table with respect to the imaging setup. To achieve this, imaging has become an important tool in radiotherapy treatment procedures specifically in the image guided radiation therapy (IGRT) and the adaptive radiation therapy. The development of body cast and head mask system has provided a non-invasive patient fixation. However, the interfractional and intrafractional anatomy change of the patient cannot be detected by any means of the non-invasive method.^4^ Also; the daily changes in human body alter the soft tissue landscapes within a patient’s anatomy, which further result in more gradual changes in target and other related structures during the course of the radiation therapy.

Mega voltage CT (MVCT) imaging on TomoTherapy Hi-ART (TomoTherapy Inc. Madison, WI) machine, mega voltage and kilovoltage cone beam computerized tomography (CBCT) on Varian linear accelerator with mobile C-arm kilovoltage imager are often used to keep track of the anatomical changes, taking place during the treatment. This raises the probability of dose conformation in the target region and decreases the severity of side effects. For some cases, acquiring electronic portal images frequently, prior to delivery of each fraction usually does the assessment of positional changes and registering these images with digitally reconstructed CT data.^5^ Corrections are made on the basis of the significance of differences. But the simple translation cannot capture the full extent of anatomic changes, as organs are not rigid. The actual deformation depends on the changes in shape as well as location of the OAR or target and hence is 3-dimensional (3D) in nature. The changes are thus not fully accessible with the simple translation.

**FIGURE 1:**
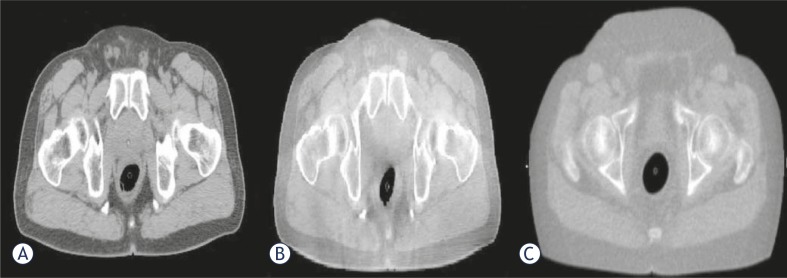
(A) kVCT, (B) kV CBCT and (C) MVCT images in transverse view are represented. As seen in figure the soft tissues contrast is better for kV CBCT images in comparison to MVCT image.

CBCT adequately provides the volumetric data of target and surrounding anatomical structures (bones and soft tissue).^6^–^8^ The 3D kV CBCT systems are extensively used in IGRT for patient setup, visualization and localization. Multiple vendors have installed on board imagers using kV X-ray on linear accelerators. Onboard imager helps to resolve the critical aspects of IMRT such as patient setup and target localization.^9^ A CBCT image of the patient can be acquired in about 60 seconds just before the delivery of each treatment fraction. The CBCT using a kilovoltage imaging system mounted on a linear accelerator has emerged as a significant technique for realising the soft tissue registration.^10^ Presently, adaptive planning is frequently done on MVCT and kV-CBCT images to conform the dose distribution and dose coverage to the target and OAR due to significant weight loss during the treatment, shrinkage in tumor or re-growth of the tumor volume. For any sort of the adaptive planning, it is important to make use of correct parameters like image set and CT to density table.^11^ Doing adaptive planning on the regular basis requires a routine check of the machine’s CT to density curve. The changes in the CT to density curve are introduced due to the variation in the Hounsfield number (HU) or CT number. Richter *et al.* (2008) have studied that the mean difference of 564 HU ± 377 HU was observed in the CT values of the CBCT image and CT image. Thus, it becomes important to check for the stability of the CT to density curve and its effect on the planning. Also, for the adaptive planning, it is important to have a good images quality. Figure 1 shows that for soft tissues, the kV CBCT images are superior to the MVCT images, thus making re-contouring is easier on kVCT images. The adaptive radiotherapy treatment is practically helpful. Real time intrinsic anatomical imaging data, dose calculations using valid CBCT numbers and necessary amendment in plan on the basis of the revised target and OARs localization are primary requirements for it.^12^–^14^

Seco and Evans in 2006, observed that the use of electron density, rather than mass density, at the treatment planning system gives better precision in dose calculations.^15^ Also, Morin *et al.* in 2006 investigated the feasibility of using MV CBCT for the dosimetric impact of changing anatomy and subsequently applying it for the adaptive radiotherapy.^16^ Yang *et al.* (2007) investigated the effect of scatter on the reconstructed CBCT pixel values and found that CBCT image reconstruction of a transverse slice was dependent on the scatter through the entire volume.^17^ So, adequate phantoms (earlier only 5 cm long phantom were used and so consequently had less scan volume) should be used for HU number to the electron density calibration for real patient imaging. At present, there is a lack of literature which can elucidate the details of the precisions and limitations of HU to the electron density calibration of kV CBCT over the longer duration of time and this limitation hampers the utilization of capabilities of this technology for the adaptive radiotherapy.

The overall purpose of this paper is to study the stability of the CT to density curve of kV CBCT imaging modality over the period of six months using electron density phantom and subsequently, analyzing the effect of CT to density curve in the clinical plans. We have investigated the feasibility and usefulness of kV CBCT for the adaptive radiotherapy and the dosimetric aspects with respect to volumetric changes and calibration curve are analyzed.

## Materials and methods

“Trilogy” (Varian) offers a broad range of external beam energy for the treatment of palliative cases, 3D conformal radiation therapy (CRT), and IMRT with multiple dose rate options. It is a comprehensive delivery system built on the foundations of Clinac iX platform. Along with the capabilities of a Clinac 21EX, the Trilogy accelerator has an extensive list of new features like Stereotactic mode (6 MV beam, up to 1000 MU/min dose rate, up to 6000 MU/field total dose, 60 MU/deg dose rate for arc-based treatments and maximum field size of 15cm * 15cm), remote couch motion, 0.75 mm radius isocenter for all three rotational axes and 0.5 mm radius isocenter for gantry and collimator axes. Instant imaging is obtained with the help of the portal imager. On-Board Imager, kV imaging system is standard on Trilogy linear accelerator which makes dynamic targeting IGRT more efficient and convenient. During this work, we have used this machine. The stability of the CT to electron density calibration is an indicator of the CT number integrity and a prerequisite for the dose recalculation. To study the stability of CT to the electron density table, the electron density phantom was used.

The electron density phantom (Fluke model 76-462) is composed of an inner head and outer torso section and has a series of inserts of known physical densities. The phantom is manufactured from durable epoxy and the tissue equivalent plugs and can be positioned at 17 different locations within the scan field. The phantom has the inserts for the breast, lung (inhale and exhale), liver, dense bone, muscle, adipose and trabecular bone. A distance registration can be quickly accessed with special marker plugs. The CBCT scans were acquired with an on-board imager system mounted on a Trilogy linear accelerator (Varian Medical Systems). The scan duration was 60 seconds with 640 projections acquired over 360 degrees. The “Electron Density” phantom was scanned multiple times over a period of six months. The kV CBCT images acquired were then imported to the Pinnacle 8.1 treatment planning system (TPS) (Philips Medical Systems, Fitchburg, WI) to measure image values. Regions of interest were contoured at the centre of each of the phantom plugs and the mean HU values within the contours were recorded. The electron densities of each phantom plug were recorded from the manufacturer specifications and the physical density corresponding to the mean CT values was recorded and plotted as the CT to density curve. The CT to density curve was recorded for a period of six months by scanning the phantom twice a week.

The effect of variations in CT to density curve was studied on two clinical cases: prostate and lung. The lung was treated using 3D CRT and the prostate by using an IMRT treatment plan. The lung tumor was prescribed to 52 Gy in 2 Gy per fraction for the treatment and the prostate was prescribed to 70 Gy in 2.5 Gy per fraction. Each case had one kVCT image called reference image which was used as a planning CT. kV CBCT images were acquired for registration purposes. A treatment plan with the appropriate target coverage and minimum dose to sensitive structures was planned on kVCT. The dose distribution based on the daily kV CBCT images was calculated using Pinnacle 3 8.1 treatment planning system (TPS) (Philips Medical Systems, Fitchburg, WI). The kV CBCT images were exported to Pinnacle planning station and the images were aligned with the kVCT images using fusion tools. Dose re-computations for each case were recalculated for the acquired CT to density curves. Changes in the dose volume histograms (DVHs) and the dose distribution due to changes in CT to density were used to compare the plans. DVH points, such as D90 (dose to 90% of target volume), D95 (dose to 95% of target volume) for the planning target volume (PTV) and the D50 (dose to 50% of target volume) and D30 (dose to 30% of the target volume) for the critical structures were calculated and analysed for all the plans generated using different CT to density tables.

## Results

**FIGURE 2:**
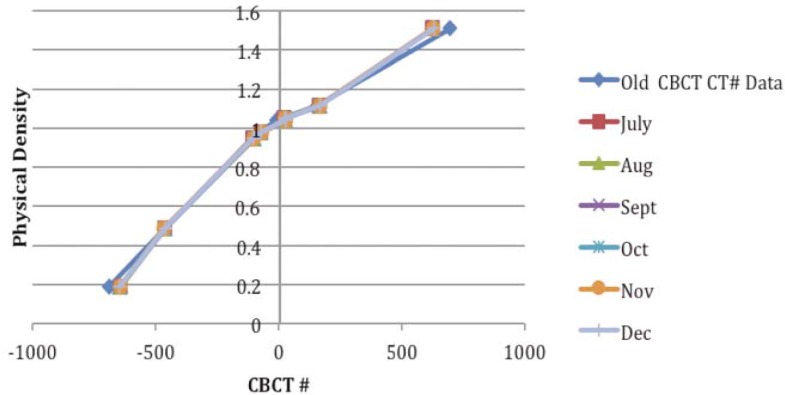
CT to density curve generated over a period of six months using electron density phantom.

**FIGURE 3:**
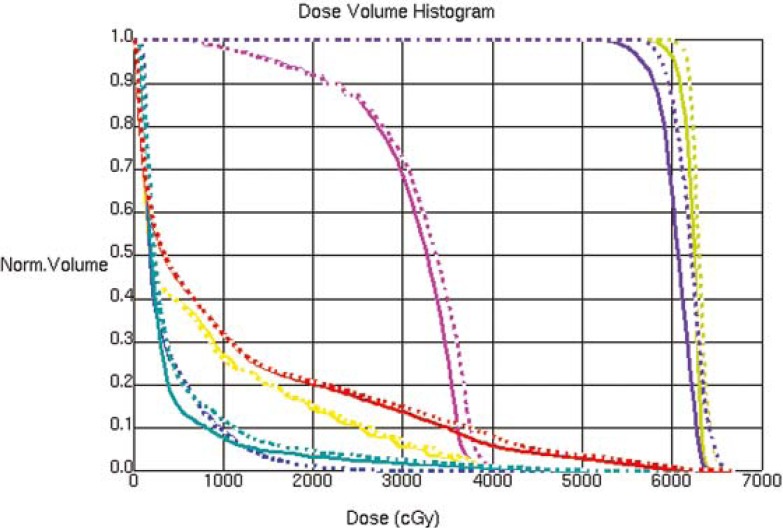
Dose volume histogram for the lung case using the original CT to density curve, for the month of October and November.

**FIGURE 4:**
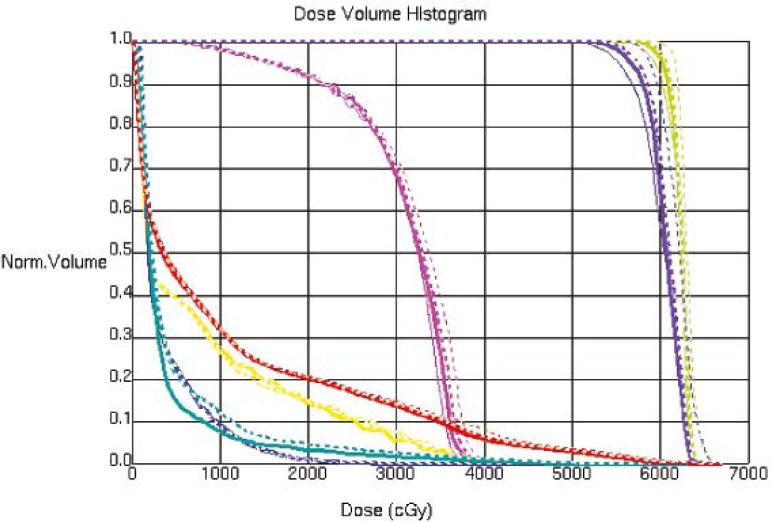
Dose volume histogram for the lung case using CT to density curves generated for last four months.

The CT image used for the treatment planning and the kV CBCT image acquired on one of the treatment day are shown in Figure 1, an MVCT image is also shown for the image quality comparison. The kV CBCT images have good soft tissue visibility and can be used for the contouring if required. The CT to density curve is generated over a period of six months for the kV CBCT using the electron density phantom. The CT to density curve for the CBCT images is fairly consistent. As seen in Figure 2, a small percentage difference of 3% is observed in comparison to the original used kV CBCT, CT to density curve. This percentage difference was consistent in the consecutive months. In order to analyze the effect of this difference on the clinical plans, kV CBCT acquired for the patient set were exported to Pinnacle and images were registered to the primary planning kVCT scan using the Syntegra automatic registration software operating within Pinnacle. Figure 3 shows the difference in the DVH for lung case generated using the original CT to density curve and the CT to density curve generated for the month of November. Figure 4 shows the DVH results using the CT to density curve for last four months. It was noted that for the entire analysis period, there was no significant variation in calibration curve for all physical densities and also does not show a significant difference in the clinical DVHs.

**FIGURE 5:**
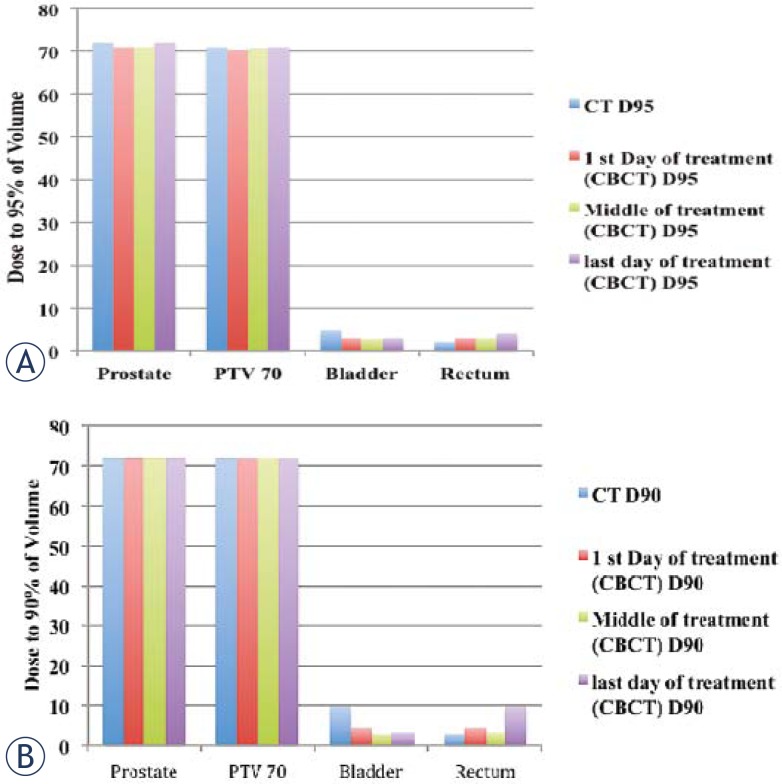
Histogram representing the dose received by the (A) 95% and (B) 90% of volume for prostate PTV70, bladder and rectum on the first, middle and last day of the treatment and for day of kVCT scan and original CT scan.

**FIGURE 6:**
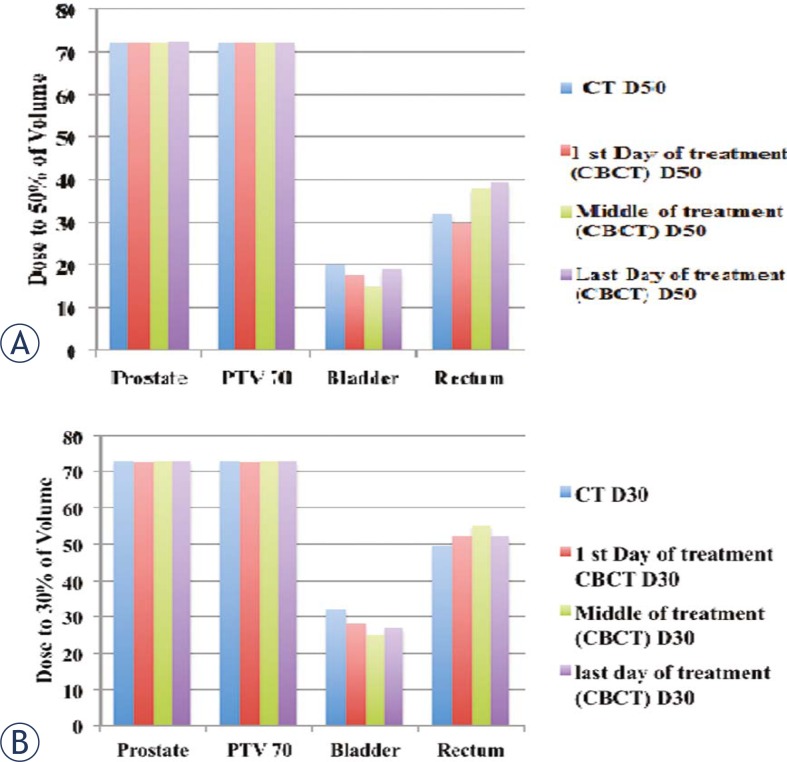
Histogram representing the dose received by the (A) 50% and (B) 30% of prostate, PTV70, bladder and rectum volume on the first, middle and last day of the treatment and for day of kVCT scan and original CT scan.

**FIGURE 7:**
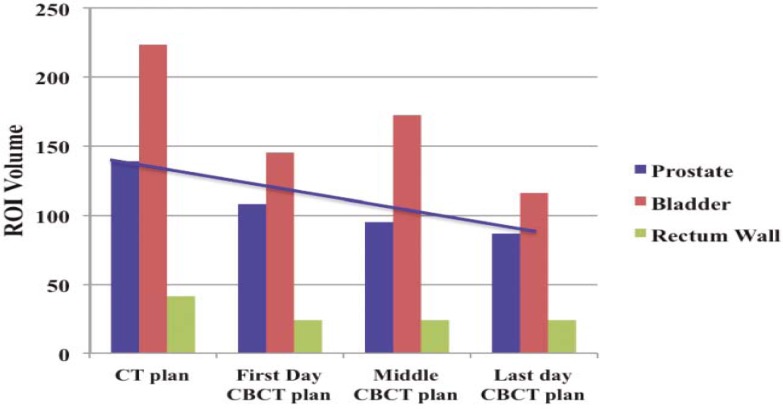
Histogram shows the variation in the volumes of prostate, bladder and rectum wall on, first, middle and last day of the treatment and for day of kVCT scan. The units of ROI volumes are represented in cc.

For the prostate case, the dose to the prostate remains fairly alike for all treatments but the dose to the bladder and rectum shows a considerable variation between the first and last day of the treatment for D90 (dose received by 90% of the volume) and D95 (dose received by 95% of the volume) as shown in Figure 5 (a, b). Figure 6(a, b) summarizes the results for D30 and D50. For better comparison the results from the original plan (kV CT based plan) is also displayed. The PTV volume showed a slight variation on day-to-day basis. Similar trend is observed for the bladder but there is less variation in the rectum volume. The results for the variation in the volumes of PTV, bladder and rectum wall are represented in the histogram format in Figure 7. It is observed that the patient’s bladder was systematically smaller on all CBCT scans compared with the planning CT scan. In order to investigate the deviation in dosimetry for the treatment period, we also analyzed the standard deviation for the entire treatment period. It has been observed that there is a negligible standard deviation in the daily prostate mean dose in kV CBCT plan as well as in case of kVCT plan. The relative standard deviation of 0.5 is observed for PTV (PTV70) in kV CBCT plan and kV CT plans. On the other hand, the relative standard deviation of 4.5 in case of bladder and approximately 2 for rectum are noticed. The relative standard deviation in daily dose to 50%, 30% and 20% of bladder is around 4 to 4.3 for CBCT plan and likewise, for a daily dose to 50%, 30% and 20% of rectum wall is 2.3, 2 and 3.9, respectively. The difference in the dose received by the 90%, 95%, 50% and 30% volume of the PTV, bladder and rectum was not due to the CT to the density table but was mainly due to the displacement of the prostate due to the variation in the bladder and rectum filling on each day.

## Discussion

Adaptive radiotherapy is an evolving area of much interest, aimed at developing techniques by which a course of radiation therapy could continually be monitored and modified to reflect the anatomic changes known to occur. So, acquiring dose information at radiation treatment course time serves as feedback necessary for the re-evaluation and subsequently adjusting the plan if necessary to account for discrepancies, anatomical changes and variation in the tumor size is a critical part of the adaptive radiotherapy. The CT to density curve for the kV CBCT shows very slight changes over the period of six months. Thus, the adaptive planning on the kV CBCT images can be performed without generating a new CT to density curve as required for the adaptive planning on MVCT images. Studies show that the dose difference of 5% can be observed if an incorrect image value to density table is used for the adaptive planning for MVCT. CBCT helps in attaining a prudent and efficient plan, as over the entire treatment period no system related dosimetric discrepancies are observed and also the same CT to density curve is functional over the entire length of the treatment. The difference in the dose to the rectum and bladder are due to the lack of reproducibility of the daily set up for the prostate treatment. Hence, it is important to perform the adaptive planning to check the dose to the target and OAR, if possible without performing a new CT scan, which will reduce the dose to the patient. Also, the image quality of the kV CBCT images are superior for the soft tissues for contouring. Usually, using the large margin for the PTV can help to compensate for setup errors but this may not be of great help for the prostate cases since the rectum and bladder volume shows the variation on each day of the treatment. Also, by increasing the PTV, the dose to bladder and rectum may increase. The presented study confirms consistently the variations in tumor (both shape and size), and bulk of soft tissue information for daily image guidance. Significance of dose difference of 5% to 6 % or more on the single day of the treatment is beyond the scope of this study.

## Conclusions

CBCT based imaging is a preferable option for the anatomical delineation of soft tissues due to its superiority in comparison to MVCT based imaging. From the perspective of the adaptive planning, this system improves the overall workflow with a quick review and analysis of the dynamic requirements. The acceptability of the same CT to the density curve is ascertained by the stability of the curve for the adequate treatment period. Though there might be variations to be accommodated in the treatment plan, the main reason behind such variations is due to differences in anatomy over time. Thus, kV CBCT has potential to act as a valuable tool for adaptive radiotherapy and significantly helps in avoiding the excessive patient scanning which may lead to cumulative high doses in patients.
